# The impact of the COVID-19 pandemic on cancer care including innovations implemented in Sub-Saharan Africa: A systematic review

**DOI:** 10.7189/jogh.13.06048

**Published:** 2023-11-17

**Authors:** Sarah Rine, Shana T Lara, Jean C Bikomeye, Sara Beltrán-Ponce, Solomon Kibudde, Nixon Niyonzima, Olatunji O Lawal, Pius Mulamira, Kirsten MM Beyer

**Affiliations:** 1 Division of Epidemiology & Social Sciences, Institute for Health and Equity, Medical College of Wisconsin, Milwaukee, Wisconsin, USA; 2 Department of Radiation Oncology, Medical College of Wisconsin, Milwaukee, Wisconsin, USA; 3 Uganda Cancer Institute, Kampala, Uganda; 4 Department of Obstetrics and Gynecology, University College Hospital, Ibadan, Nigeria

## Abstract

**Background:**

The coronavirus 2019 (COVID-19) pandemic has caused disruptions in the delivery and utilisation of cancer services. The impact of these interruptions is disproportionately borne by low- and middle-income countries in Sub-Saharan Africa (SSA). There are speculations of increased late-stage presentation and mortality as services are returning to the pre-pandemic state. This review aims to explore the extent to which the COVID-19 pandemic impacted cancer services across SSA and to identify innovations implemented across SSA to mitigate the impacts.

**Methods:**

Using database-specific search strategies, a systematic literature search was conducted in PubMed, Ovid (MedLine), Web of Science, and African Index Medicus. Eligible studies included original research, reports, perspectives and summaries of national or regional outcomes published in the English language. The primary outcome was changes in the delivery and utilisation of cancer prevention and screening, diagnosis, treatment and follow-up services. The secondary outcome was to identify implemented innovations to mitigate the impact of the pandemic on service delivery.

**Results:**

Out of the 167 articles identified in the literature search, 46 were included in the synthesis. A majority (95.7%) of the included articles described suspension and/or delay of screening, diagnosis, and treatment services, although two studies (4.3%) described the continuation of services despite the lockdown. Care was additionally impacted by transportation limitations, shortages of staff and personal protective equipment, disruption of the medication supply chain and patients’ fears and stigma associated with contracting COVID-19. A major innovation was the use of telemedicine and virtual platforms for patient consultation and follow-up during the pandemic in SSA. Furthermore, drones and mobile applications were used for sample collection, medication delivery and scheduling of treatment. In some instances, medication routes and treatment protocols were changed.

**Conclusions:**

The delivery and utilisation of cancer services decreased substantially during the pandemic. Cancer centres initiated innovative methods of care delivery, including telehealth and drone use, with long-term potential to mitigate the impact of the pandemic on service delivery. Cancer centres in SSA must explore sustainable, facility or country-specific innovations as services return to the pre-pandemic state.

**Registration:**

The review was registered in PROSPERO with registration number CRD42022351455.

The coronavirus disease 2019 (COVID-19) has significantly interrupted all aspects of medical care delivery and utilisation globally [1]. As the World Health Organization (WHO) noted: “People, efforts, and medical supplies all shifted to respond to the emergency. This often leads to the neglect of basic and regular essential health services, including those related to cancer. People with health problems unrelated to the epidemic find it harder to get access to healthcare services” [2]. These interruptions occurred across many countries, but low- and middle-income countries (LMICs) have been disproportionately affected [3,4].

In Sub-Saharan Africa (SSA), cancer is a growing public health problem. It is one of the three leading causes of premature death (i.e. death among those ages 30-70 years) in almost all SSA countries [5]. Cancer is responsible for one in seven premature deaths overall and one in four deaths from noncommunicable diseases [6]. Over 800 000 new cases of cancer and 500 000 cancer deaths were recorded across SSA in 2020 [6]. The mortality-to-incidence ratio is more than double the rates observed in high-income countries (HIC). This indicates a higher likelihood of death from cancer within SSA [7]. The cancer burden is increasing in SSA, but the healthcare infrastructure is not robustly established and is unable to respond adequately. Thus, the COVID-19 pandemic has impacted an already inadequate system for cancer prevention and control [8].

Globally, the majority of healthcare services were delayed and/or suspended secondary to the pandemic [9-11]. Additional consequences, such as the disruption of drug and equipment supply chains, travel restrictions due to lockdowns and/or curfews, stigma associated with having COVID-19, and loss of income due to COVID-19 all contributed to the underutilisation of cancer services [12-14]. Some individuals with cancer were unwilling to utilise cancer care even where available because of fear of contracting the virus [15]. Further complicating matters, immunosuppression caused by cancer and/or its treatment places individuals with cancer at risk of opportunistic infections compared to other members of the population [16,17]. This immunocompromised status increases the risk of more serious complications from COVID-19 infection among individuals with cancer [16].

There are speculations that cancer incidence, late-stage diagnosis, and mortality may increase as cancer care services return to pre-pandemic levels. This could be because of missed screening and diagnoses during pandemic spikes [17,18]. Although indications of the adverse impact of the pandemic have been reported, the impact of the pandemic on cancer care in SSA is unclear, as is the nature of the response.

This review aims to explore the extent to which the pandemic has impacted cancer care services across SSA and to identify innovations implemented across the SSA region to mitigate the impact of the pandemic on cancer prevention and control. We hypothesise that the COVID-19 pandemic caused a decrease or delay in cancer care across SSA.

## METHODS

This review is reported using the Preferred Reporting Items for Systematic Reviews and Meta-Analyses (PRISMA) 2020 guidelines. The PRISMA extension checklist was used as a reporting guide. The review was registered in PROSPERO with registration number CRD42022351455. PROSPERO is the international prospective register of systematic reviews in health and social care. It compiles a listing of protocols of systematic reviews in an attempt to avoid duplication of effort, reduce reporting bias and promote transparency [19].

### Literature search and selection criteria

A literature search was conducted in collaboration with an institutional librarian. A systematic literature search was conducted on 10 August 2022, in PubMed, Ovid, Web of Science, and African Index Medicus, limited to articles published in English. The search was limited to these databases because they index a large range of articles; therefore, providing a more comprehensive picture and minimising selection bias.

The search was conducted using medical subject headings (MeSH) and keywords combined with database-specific search techniques. A list of MeSH headings and Keywords is presented in Table S1 in the **Online Supplementary Document**. Articles were exported from search engines into EndNote for de-duplication and then uploaded into Rayyan [20], an online tool useful for article screening and selection.

The eligibility criteria were determined in advance and were relevant in the screening and selection of articles. Studies were included if they were original studies, reports, perspectives, recommendations or summaries of national or regional outcomes of the COVID-19 pandemic on cancer care delivery and utilisation. Other criteria included: human studies, published in the English language, full-text availability, publication in or after 2020 and studies from or describing SSA. These inclusion criteria were selected to decrease ambiguity and to ensure that no study was excluded without a thorough screening.

### Article selection, data extraction and analysis

 SR and STL screened all retrieved articles to determine eligibility. Blinded screening for titles and abstracts was conducted in Rayyan with conflicts were resolved following a discussion and group consensus.

The full-text analysis focused on the outcome and impact of the COVID-19 pandemic on cancer care. This was subcategorised into themes as follows: impact on prevention and screening services, impact on diagnostic services, impact on treatment (including surgery, endocrine therapy, radiotherapy, chemotherapy), impacts on follow-up and innovations set up by facilities and/or the government. An additional category for “other” factors with impact on cancer services was also analysed to ensure no relevant data was uncaptured. Information for each theme was extracted from the text systematically and categorised as suspended/delayed or no change. Findings were also subcategorised according to the World Bank classification of the SSA sub-regions: Central, Eastern, Southern and Western Africa.

Extracted data from all included studies were summarised in a MS Excel (Microsoft Inc, Seattle WA, USA) data set presented in Table S2 in the **Online Supplementary Document**. The resulting Excel data set and the RAWGraph [21] tool were used to generate an alluvial chart, something adopted from previous work [22,23] in reporting trends across findings when multiple studies in a review are considered altogether. Studies with no reports on preventive services, diagnosis, treatment and follow-up were excluded from the alluvial chart; but those with partial reports were included.

### Quality assessment

Most included articles were perspectives or recommendations and descriptive. Such designs are prone to biases that should be thoughtfully considered when interpreting results. A few studies were cross-sectional and cohort. Therefore, each article was assessed for potential bias types, sources of funding and conflicts of interest. For any article without quality assessment, a reason was stated. SR and RCR conducted the bias assessment. The Newcastle-Ottawa scale was used for both cross-sectional and cohort studies, and the quality assessment tool developed by the National Heart Lung and Blood Institute (NHLBI) was used for assessing bias for pre/post studies without a control group. Each article was reviewed by two independent reviewers and scores were provided according to the scales used. An average score was provided following the assessment and the article was classified as poor, fair, and good. Descriptive and retrospective studies were assessed individually. These details are presented in Tables S3 to S6 in the **Online Supplementary Document**.

## RESULTS

### Articles selection and characteristics of included studies

**Figure 1:**
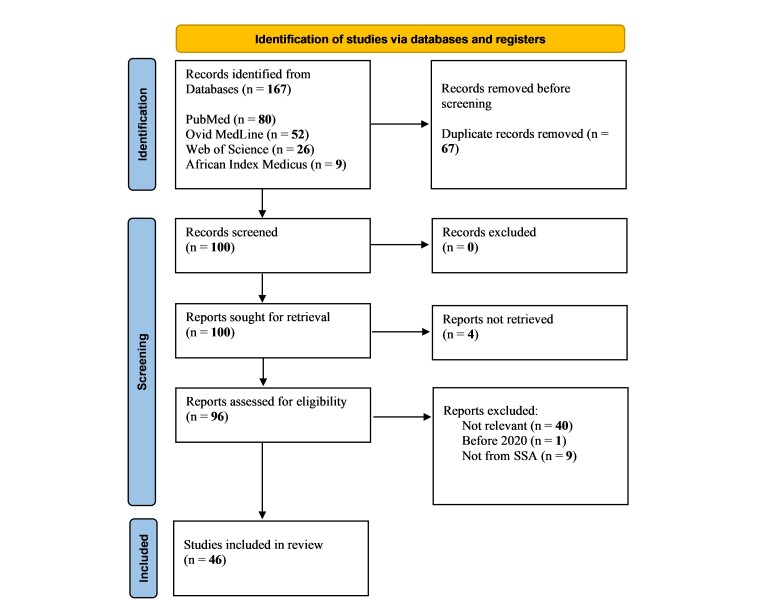
A graphical representation of the PRISMA guidelines adapted from Page et al. for the selection process of articles included in the review [24].

A total of 167 articles were retrieved after the original database search. A total of 100 articles remained after excluding duplicates, four of which were excluded because reports were not retrieved. Therefore, 96 articles were eligible for screening, 50 of which were excluded for various reasons leaving a total of 46 studies included in the review. **Figure 1** describes the article selection flowchart adapted from Page et al. [24].

The total of 46 included articles spanned across countries in several SSA sub-regions:15 from Eastern Africa, 13 from Southern Africa; 10 from Western and Central Africa; five were multi-national; and three articles described combined Western, Eastern and Southern regions. **Table 1** describes the characteristics of all included studies.

**Table 1:** Characteristics of included studies

Author	Country	Cancer type	Study design	Sub-theme(s) covered
Abila et al. [25]	Uganda	N/A	Expert commentary	Screening, treatment, follow-up, other indirect factors
Bhutta et al. [26]	South Africa	Head and neck	Descriptive study	Innovations
Chu et al. [27]	South Africa	Breast and colorectal	Retrospective analysis	Treatment: surgery
Chu et al. [28]	South Africa	N/A	Cross-sectional survey	Treatment: surgery
Davey et al. [29]	Botswana	Gynaecologic	Descriptive study	Diagnosis, follow-up, innovations
Benn et al. [30]	South Africa	Breast	Descriptive study (letter to the editor)	Treatment: surgery innovation
Desta et al. [9]	Ethiopia	Cervical	Pre-post intervention study	Screening, treatment
Diao et al. [31]	Zambia	N/A	Pre-post intervention study	Innovations
Ezenwankwo et al. [32]	SSA wide	Prostate	Descriptive study	Diagnosis, treatment
Ezenwankwo et al. [33]	West Africa	Prostate	Descriptive study	Screening, diagnosis, treatment, innovations
Haddison et al. [34]	Cameroon	Cervical	Cross-sectional survey	Prevention
Henke [35]	East Africa	N/A	Descriptive study (letter to the editor)	Screening, diagnosis, treatment, indirect factors
Irusen et al. [36]	South Africa	Prostate	Cross-sectional survey	Indirect factors
Joseph et al. [37]	Nigeria	Many	Cross-sectional survey	Treatment
Kabukye et al. [38]	Uganda	N/A	Mixed methods study (qualitative: focus group discussion and interviews)	Innovation
Karanja-Chege [39]	Kenya	Cervical	Descriptive review	Prevention
Lombe [40]	Zambia	N/A	Descriptive review	Treatment, follow-up, innovation
Martei et al. [41]	SSA wide	N/A	Cross-sectional survey	Treatment, innovation, other factors
Masege et al. [13]	South Africa	Ear, nose and throat	Descriptive review	Diagnosis, other factors
Moustakis et al. [42]	South Africa	N/A	Retrospective analysis	Treatment
Muli et al. [43]	Kenya	N/A	Intervention study	Innovation
Murewanhema [12]	Zimbabwe	Cervical	Perspective review	Screening, other factors
Murewanhema [11]	Zimbabwe	Cervical	Perspective and recommendation	Treatment, other factors
Murewanhema [44]	Zimbabwe	N/A	Perspective and recommendation	Treatment, follow-up
Ngwa et al. [45]	SSA wide	N/A	Perspective and recommendation	Innovation
Olabumuyi et al. [46]	Nigeria	N/A	Expert panel discussion	Treatment, other factors
Osibogun et al. [47]	Nigeria	N/A	Retrospective analysis	Other factors
Ramdas et al. [48]	South Africa	Breast	Retrospective analysis	Treatment
Salcedo et al. [49]	Mozambique, Angola, Zambia	Cervical	Descriptive review	Innovation
Weber et al. [50]	Rwanda	Gynaecologic	Perspective review	Innovation
Villain et al. [10]	Rwanda, Cote d’Ivoire, Cameroon, Zambia	N/A	Cross-sectional survey	Screening, diagnosis, treatment, innovation
Van Wyk et al. [51]	South Africa	Breast, cervical, esophageal, stomach, colorectal, prostate	Retrospective analysis	Diagnosis
Umutesi et al. [52]	Rwanda	N/A	Descriptive	Diagnosis, treatment, follow-up, other factors
Umar et al. [53]	Kenya	N/A	Cross-sectional survey	Diagnosis, treatment, other factors
Sumbana et al. [54]	Mozambique	N/A	Descriptive	Other factors
Sormani et al. [55]	Cameroon	Cervical	Descriptive	Screening
Sikakulya [56]	DR Congo, Uganda	N/A	Cross-sectional survey	Other factors
Okunade et al. [57]	Nigeria	Gynaecologic	Perspective and recommendation	Diagnosis, treatment, follow-up, innovation, other factors
Selgado et al. [58]	Ethiopia	N/A	Retrospective analysis	Other factors
Rosa et al. [59]	Liberia	N/A	Descriptive	Other factors
Argefa [60]	Ethiopia	Cervical	Prospective cohort study	Other factors
Kugbey et al. [4]	Ghana	N/A	Descriptive	Diagnosis, treatment, other factors
Kassaman et al. [61]	Kenya	N/A	Descriptive	Other factors
Salako et al. [62]	Nigeria	N/A	Descriptive	Treatment
Okeke et al. [63]	Africa wide	N/A	Descriptive	Innovation, other factors
Vanderpuye et al. [64]	West Africa, South Africa, Sudan	N/A	Descriptive	Treatment, innovation

N/A – not reported

### Impact on preventive and/or screening services

Prior to the pandemic, many SSA countries lacked comprehensive cancer screening infrastructure. Generally, the minimal infrastructure in countries with screening services is inadequate to serve the population, often leading to low screening coverage and rates, especially in rural settings [65,66].

While a majority of the countries experienced suspension of screening and preventive services, some countries were able to continue routine services, even during lockdown periods. In most of Western and Central Africa, prostate cancer awareness programmes were suspended [33]. In Cameroon, cervical cancer screening services were also suspended [10,55]. The crude number of women screened for cervical cancer in Cameroon in 2020 decreased by almost 80% compared to 2019 [55]. The pandemic also contributed to hesitancy in the uptake of the Human Papillomavirus (HPV) vaccine [34]. An HPV vaccination programme for adolescents in Cameroon was introduced during the acute phase of the pandemic. The community believed that the vaccine was being used as a cover by pharmaceutical companies to infect their children with the coronavirus, which served as a significant barrier to the uptake of the vaccine [34].

Similar findings were seen in East Africa [35]. In Kenya, there was a complete disruption of HPV vaccination programmes. In Uganda, screening services and cancer awareness outreach programmes were all suspended [25,39]. In Ethiopia, there was a 54.8% decrease in the crude number of women aged 30 to 49 years screened for cervical cancer during the early phase of the pandemic compared to the previous year [9]. Zimbabwe and Zambia also reported suspension of screening services, and despite the easing of lockdown and curfew, there are reportedly minimal services ongoing in both nations [10,12]. Few countries had only minor disruption to preventive or screening services. For example, in Cote d’Ivoire and Rwanda, screening was ongoing for colon cancer, although on a smaller scale [10].

### Impact on diagnostic services

The majority of countries in SSA suspended, cancelled or scaled down diagnostic services, especially during the lockdown. In Botswana, there was a significant decrease in the number of patients who attended new diagnosis appointments during the lockdown compared to before the pandemic [29]. An Ethiopian study also revealed an 84.2% decrease in the crude number of women aged 30 to 49 years who were diagnosed with cervical cancer [9]. Nigeria and Ghana, both West African countries, also recorded delays, cancellations and suspension of diagnostic evaluations for gynecological cancers [4,57]. In a report that included several SSA countries, there was a delay in the diagnosis of prostate cancer [33]. Similarly, many countries in West Africa recorded significant delays in scheduling diagnostic procedures for prostate cancer [32,33].

In South Africa, the crude number of patients who presented for staging pan-endoscopy for head and neck cancers decreased by 50% during the lockdown [13]. Additionally, the overall histopathology case load decreased by almost 50% [51]. New pathological diagnoses for six selected cancers combined (breast, prostate, cervical, colorectal, esophageal and stomach) decreased by 36.2% [51]. In Kenya and Rwanda, there were delays in scheduling diagnostic procedures, especially for new patients [52,53].

### Impact on treatment services

#### Eastern Africa

There was a significant disruption in treatment services. The COVID-19 pandemic worsened already limited access to cancer care in SSA. In Uganda, there was poor adherence to treatment schedules and delays in scheduling and initiating cancer treatments [25]. In Ethiopia, there was a recorded 85% decrease in the number of women aged 30 to 49 years receiving treatment for cervical cancer [9]. In Uganda, Kenya, and Tanzania, there were interruptions of ongoing cancer treatments. Treatment plans were delayed, and individuals with cancer experienced difficulties in accessing supportive medications like analgesics and anti-emetics [35,53]. Rwanda similarly experienced limited access to treatment services and refilling of other medications was suspended [52]. In Sudan, elective surgeries and non-urgent intravenous chemotherapy were suspended [64].

Before the pandemic, radiotherapy was conducted on a first come first serve basis in Zambia [10], however during the lockdown, radiotherapy sessions were scheduled with time slots allocated to patients, which decreased the number of patients seen in a day. Radiotherapy protocols were adjusted to decrease treatment course and duration [10]. Similarly, chemotherapy was scheduled to avoid crowds in treatment areas [40]. Surgical services were delayed for patients whose procedures could be deferred after triage. Non-surgical alternatives like hormonal therapies were considered wherever possible, and surgical procedures were decided on a case-by-case basis with an overall decline [40].

#### Southern Africa

Findings regarding treatment in South Africa were mixed. In a survey of 85 hospitals, 61 (71.8%) of those continued all cancer surgeries despite the lockdown, although patient volume decreased. A total of 21 hospitals (24.7%) continued only symptomatic cancer surgeries and three hospitals (3.5%) cancelled all cancer surgeries [28]. Another South African report indicated that the frequency of surgeries for breast and colorectal cancers did not change significantly [27]. The TARGIT-Intraoperative Radiotherapy (IORT) protocol or treatment frequency remained the same for breast cancer [48]. A different study indicated that there was a decrease in surgeries conducted for breast cancer in other facilities in South Africa [42]. In Zimbabwe, all treatments were completely halted during the lockdown [44].

#### Western and Central Africa

A survey of 1072 individuals with cancer in Nigeria indicated that there were disruptions in service delivery with 17.4% reporting disruptions in cancer care and 9.8% and 9.7% reporting cancellation of radiotherapy and chemotherapy appointments respectively. Ten percent reported a change of route of administration of chemotherapy from injections to oral medications [37]. Another Nigerian report noted that both chemotherapy and radiotherapy were scaled down or suspended in some centres and changes were made to the existing surgical protocols [46,62]. In Cameroon, non-urgent cancer treatments were suspended while screening services and diagnostic testing for screen-positive individuals continued in Cote d’Ivoire [10].

Non-country specific reports indicated a delay in treatment for prostate cancer [32,33]. In a multi-country cross-sectional survey of professionals, 71.4% of respondents reported the use of specific treatment guidelines with 55.7 and 48.6% of those being institutional-based guidelines and guidelines from the Ministry of Health [41]. Thirty percent of respondents reported that individuals with newly diagnosed cancer experienced delays in treatment initiation [41]. One-third of respondents reported a change in treatment protocols. The majority of changes were delays in palliative treatment, adjuvant chemotherapy, and curative radiotherapy. Other changes were related to the use of hypofractionated or ultra hypofractionated radiotherapy [41].

### Impact on follow-up

New patient appointments continued, but follow-up appointments for existing patients were cancelled in Botswana [29]. Similarly in Zambia, all new patients were prioritised for the establishment of treatment plans while existing patient visits were conducted via telephone [40]. Outpatient follow-up clinics for new and existing individuals with cancer were closed early in the pandemic. Outpatient follow-up was significantly scaled down in Zimbabwe and Nigeria [44,46]. In-person follow-up was minimised and suspended in some facilities in South Africa and Rwanda [52,57,64]. In Rwanda, a weekly patient list was generated from the electronic patient records and used to plan follow-up visits [52]. Uganda recorded an increase in loss to follow-up after cancer diagnosis [25].

### Other factors that impacted cancer care

Several additional factors were identified as contributing to disruption in cancer care. Public transportation was restricted in many locations because of regional or national lockdowns and curfews. Transportation companies doubled costs to consumers in order to cover the cost of reduced carrying capacity [25,35,46,57]. The restrictions on economic activities further exacerbated financial constraints, making it difficult for patients to cover the cost of their treatments [13,25,53].

Many patients had a heightened fear of contracting COVID-19 and were therefore unwilling to utilise services available in the cancer centres [12,13,41,46,61,63]. In some communities, individuals who tested positive for COVID-19 were stigmatised. The fear of stigma contributed further to the non-utilisation of services [11].

Many facilities scaled down their services, including reduced working hours. Operating hours were cut to a few hours per day and fewer patients were accommodated in limited schedules. This exacerbated pre-pandemic difficulties in assessing care [35,61]. There was a disruption of research activities and telemedicine approaches implemented in some areas were met with poor internet connections and erratic power supply [12,41].

The pandemic worsened malnutrition among a cohort of women treated for CC and this is speculated to increase cancer mortality [60]. There were documented shortages of personal protective equipment (PPE) and staff in cancer centres because many staff were redirected to participate in COVID response and care [4,12,41,56]. Further, there was documented staff burnout and a risk of total shutdown of services [67]. Significant disruptions in supply chains leading to equipment and medication shortages were documented which additionally contributed to delays in screening, diagnosis and treatment [12].

Anxiety among individuals with cancer was documented. A study from a cohort of patients treated for prostate cancer indicated that there is a positive correlation between anxiety about the pandemic and cancer outcomes [36]. In this cohort, individuals with higher income who were receiving prostate cancer treatment had more anxiety about contracting COVID-19 [36]. Evidence has shown that the risk of death is higher among individuals with cancer who contract COVID [47,54,58].

### Innovations implemented

To curtail the impact of the pandemic on cancer care, many facilities across SSA implemented innovations, some of which have the potential to provide long-term benefits. Some of these innovations only existed skeletally prior to the pandemic and were augmented during the pandemic, while others were implemented specifically to fill gaps created by the pandemic.

#### Eastern Africa

To address the effect of the pandemic on cancer research, Zambia and Mozambique formed a virtual capacity-building training that was conducted via video conferencing, leading to the development of new research and treatment protocols [31]. Teleconferencing was used to discuss research initiatives, patient treatment and follow-up [31,49]. In Rwanda, continuing medical education was moved to a virtual platform. Virtual trainings were provided for gynecologic oncology management, including modifications to treatment protocols [50].

All clinical and patient management meetings were moved to a virtual platform [40]. In Uganda, the Uganda Cancer Institute developed cancer awareness messages that were deployed using an interactive voice response system over telephone calls [38]. A mobile app was created in a Kenyan facility to enable tracking of referred patients; care providers were able to provide feedback about care via the mobile app [43]. Kenya and Rwanda initiated the use of courier services for cancer medication delivery [41]. Drone delivery systems were used for sample collection and medication delivery [10,41].

Provisions for transport services and/or reimbursement of transportation fees were initiated. In Zambia, this service was provided to individuals who had positive screening tests and these individuals were sent a short message reminder for future appointments via text messaging; the focus was to reorganise existing screening services [10]. During the pandemic in Rwanda, community outreach for cancer screening was conducted through mobile clinics [10]. Screening services were expanded to rural primary health centres to avoid overcrowding in the secondary and tertiary facilities [10]. Residential facilities, transport pick up and drop off and socio-economic support were provided to poor individuals receiving treatment at certain cancer centres [52].

#### Southern Africa

Telemedicine was adopted across many countries. Virtual consultations, case discussions and follow-up were used in South Africa [26]. Endocrine therapy was advocated for use among older patients with certain hormone-sensitive breast cancers which allowed for a long follow-up interval of approximately three months [30]. An outpatient smartphone application was deployed to schedule appointments and send automatic appointment reminders to patients in Botswana [29].

#### Western and Central Africa

Recommendations were provided for refining and revalidating existing programmes and protocols [33]. Courier services were used for medication delivery in Congo and Nigeria [41]. In Cameroon, transport reimbursement was provided to poor individuals with cancer and home-based self-sampling for human papillomavirus (HPV) testing was implemented [55].

Dedicated hotlines and/or mobile applications for individuals with cancer were created to enable appointment scheduling and follow-up virtually [10]. Telephone and/or video consultation and the use of hypofractionated radiotherapy were adopted across many facilities in West Africa [15,57,63]. In a Liberian report, virtual coaching, and check-up sessions were used to promote the well-being of staff working in a palliative facility [59].

**Figure 2:**
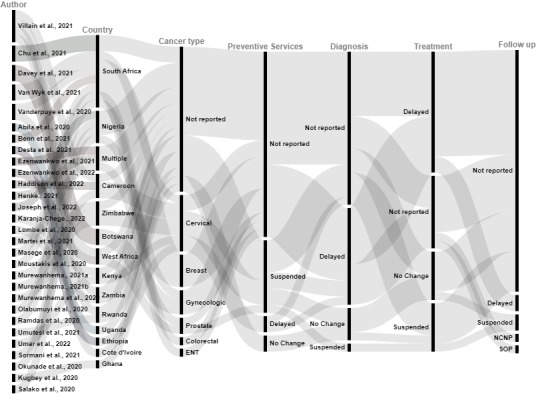
The Impact of the COVID-19 pandemic on cancer care in Sub-Saharan Africa: trends across included studies. The first column represents articles. The second column represents geographical settings of studies, including specific countries in which the studies were conducted. The third column represents diverse cancer types that were investigated in included studies. The fourth column represents impacts on preventive measures, including suspended services, delayed services, or no change. The fifth, sixth, and seventh columns respectively represent the COVID-19 impacts on cancer diagnosis, treatment, and follow-up respectively during the cancer care continuum. This graphical representation shows an overall trend in findings across all studies included. ENT – ear, nose and throat, NCNP – no change for new patients, SOP – suspended for old patients

The alluvial chart (**Figure 2**) represents a graphical illustration of trends across all our findings using studies that met additional inclusion criteria for the chart. The alluvial chart highlights the COVID-19 impacts on cancer preventive services, diagnosis, treatment and follow-up care including delays and suspension. When no information on delays or suspension was available, it was note as “not reported”. Most studies were conducted in South Africa and majority of studies did not report the cancer site of interest. Majority of studies did not report impact on preventive services, diagnosis, and follow up, while majority of studies reported delays in cancer treatment. **Table 2** provides a summary of the impact of the COVID-19 pandemic on cancer care, including other indirect factors and innovations implemented.

**Table 2:** Summary of impact of COVID-19 on cancer care (prevention/screening, diagnosis, treatment, follow-up) other indirect factors and innovations

Author	Country/Region	Cancer Types	Prevention/screening	Diagnosis	Treatment	Follow-up	Indirect factors	Innovations
Abila [25]	Uganda	-	Suspended	-	Delayed	Delayed	Transportation limitations and increased cost of transportation	-
Bhutta [26]	South Africa	Head and neck	-	-	-	-	-	Virtual consultation and follow-up
Chu [27]	South Africa	Breast colorectal	-	-	Breast cancer surgery: delayed; Colorectal cancer surgery: no change	-	-	-
Chu [28]	South Africa	-	-	-	Treatment: no change in 61 hospitals, delayed in 21 and suspended in 3 hospitals.	-	-	-
Davey [29]	Botswana	Gynaecologic	-	Delayed	-	Suspended for existing patients, no change for new patients	-	Use of smartphone and mobile application for scheduling follow-up
Benn [30]	South Africa	Breast	-	-	Surgery: delayed	-	-	Use of endocrine therapy
Desta [9]	Ethiopia	Cervical	Delayed	Delayed	-	-	-	-
Diao [31]	Zambia	-	-	-	-	-	-	Virtual capacity building and treatment protocol development
Ezenwankwo [32]	Multinational	Prostate		Delayed	Treatment: delayed	-	-	-
Ezenwankwo [33]	West Africa	Prostate	Suspended	Delayed	Treatment: delayed	-	-	Solutions to refine existing treatment programmes and protocols
Haddison [34]	Cameroon	Cervical	Human papillomavirus vaccination: delayed	-	-	-	-	-
Henke [35]	East Africa	-	Suspended	Delayed	Delayed	-	Limited public transport due to lockdown and curfews	-
Irusen [36]	South Africa	Prostate	-	-	-	-	Increase anxiety about contracting COVID-19 among patients receiving treatment for prostate cancer	-
Joseph [37]	Nigeria	-	-	-	Treatment: suspended, delayed, changes to route of medication	-	-	-
Kabukye [38]	Uganda	-	-	-	-	-	-	Cancer awareness message on interactive voice response system
Karanja-Chege [39]	Kenya	Cervical	Human papillomavirus vaccination: suspended	-	-	-	-	-
Lombe [40]	Zambia	-	-	-	Treatment: rescheduled, delayed/suspended	Patient follow-up conducted via telephone	-	Virtual patient management meetings and modification of treatment protocols
Martei [41]	Many countries	-	-	-	Treatment: delayed for new patients, radiotherapy and chemotherapy delayed, delayed surgery. Changes in the route of treatment administration	-	Staff and personal protective equipment shortages, Travel restrictions, Fear of contracting COVID-19 and financial barriers	The use of courier services for medication delivery, use of drones for sample collection, and modification of treatment protocol.
Masege [13]	South Africa	ENT	-	Delayed	-	-	Fear of contracting COVID-19, financial barriers. Travel restrictions	-
Moustakis [42]	South Africa	-	-	-	Surgery: delayed	-	-	-
Muli [43]	Kenya	-	-	-	-	The use of mobile apps to schedule follow-up visits	-	Use of mobile apps for scheduling follow-up visits
Murewanhema [12]	Zimbabwe	Cervical	Suspended and human papillomavirus vaccination suspended	-	-	-	Fear of exposure to COVID-19. Transportation restrictions	-
Murewanhema [11]	Zimbabwe	Cervical	-	-	Treatment: delayed	-	Patient factors: Fear of stigma and fear of exposure to COVID. Disruption of supply chain, personal protective equipment shortages, and travel restrictions	-
Murewanhema [44]	Zimbabwe	-	-	-	Treatment: suspended	Suspended	-	-
Ngwa [45]	-	-	-	-	-	-	-	Use of hypofractionated radiotherapy and telehealth.
Olabumuyi [46]	Nigeria	-	-	-	Surgery: suspended; radiotherapy and chemotherapy: delayed	-	Fear of exposure and travel restriction	-
Osibogun [47]	Nigeria	-	-	-	-	-	Infection with COVID-19 increase risk of cancer death	-
Ramdas [48]	South Africa	Breast			Radiotherapy: no change to targeted intraoperative radiotherapy protocol or treatment frequency			
Salcedo [49]	Angola, Mozambique, Zambia	Cervical	-	-	-	-	-	Virtual capacity building and training
Weber [50]	Rwanda	Gynaecologic	-	-	-	-	-	Virtual capacity building and training
Villain [10]	Rwanda, Cote d’Ivoire, Cameroon, Zambia	-	Rwanda and Cote d’Ivoire: no change; Cameroon: suspended; Zambia: Suspended.	Rwanda, Cote d’Ivoire, Cameroon, Zambia: no change.	Rwanda, Cote d’ Ivoire, Zambia: no change; Cameroon: suspended	-	-	Rwanda: cancer screening using mobile clinics and at rural primary health centres to avoid overcrowding at secondary and tertiary centres. Use of an online appointment application, phone calls and text reminders for patients. Cameroon: use of mobile applications for scheduling appointments. Zambia: transportation reimbursement, phone calls and text to improve scheduling
Van Wyk [51]	South Africa	Breast, Cervical	-	Delayed	-	-	-	-
Umutesi [52]	Rwanda	-	Suspended	Suspended	Suspended	-	-	Pick-up and drop-off of vulnerable patients, accommodation and feeding for individuals with cancer receiving treatment, drug refill using drones, providing social support among patients
Umar [53]	Kenya	-	-	Delayed	Delayed	-	Income reduction among patients, transportation restrictions, fear of contracting COVID	-
Sumbana [54]	Mozambique	-	-	-	-	-	Infection with COVID-19 among individuals with cancer increases risk of death	-
Sormani [55]	Cameroon	Cervical	Suspended	-	-	-	-	Transport reimbursement, use of home-based self-sampling for human papillomavirus screening
Sikakulya [56]	DR Congo, Uganda	-	-	-	-	-	Inadequate surgical preparedness because of equipment and personnel shortages	-
Okunade [57]	Nigeria	Gynaecologic	-	Delayed	Delayed	-	-	Virtual consultations and the use of hypofractionated radiotherapy
Selgado [58]	Ethiopia	-	-	-	-	-	Infection with COVID-19 among individuals with cancer increases risk of death	-
Rosa [59]	Liberia	-	-	-	-	-	-	Virtual support group meetings to promote staff well-being
Argefa [60]	Ethiopia	Cervical	-	-	-	-	Increased malnutrition among patients diagnosed with cervical cancer	-
Kugbey [4]	Ghana	-	-	Delayed	Delayed	-	Reduced income, travel restrictions, staff and PPE shortages	-
Kassaman [61]	Kenya	-	-	-	-	-	Travel restrictions, reduced working hours in cancer centres, fear of contracting COVID and associated stigma, lack of clear messaging from the ministry of health	
Salako [62]	Nigeria	-	-	-	Chemotherapy, radiotherapy, surgery: suspended	-	-	-
Okeke [63]	Africa	-	-	-	-	-	Patient’s fear of contracting COVID	The use of telemedicine
Vanderpuye [64]	SSA	-	-	-	West Africa: surgery – suspended; South Africa: no change in treatment; Sudan: surgery and chemotherapy – suspended.	South Africa and Sudan: suspended	-	Use of ultrafractionated treatment courses

## DISCUSSION

### Impact on cancer services

In SSA, the COVID-19 pandemic caused considerable disruption in cancer screening/prevention, diagnosis, treatment and follow-up services. A large percentage of the impact consisted of delays and/or suspension of services cutting across many countries, although the extent varied by country; and majority of studies included in the alluvial chart did not report the COVID-19 impact on preventive services, diagnosis and follow-up. However, trends across all studies suggest that majority of studies reported significant delays in cancer treatments across SSA. Favorable cancer outcomes are dependent on the timing of diagnosis and treatment. Several factors like government policies on lockdown and curfews, fears of contracting COVID-19, loss of income, increased treatment costs, supply chain interruptions and fears associated with stigma contributed significantly to the underutilisation of cancer services. The implications are far-reaching and contribute directly to cancer mortality.

In many countries, cancer centres were restructured to provide care to patients with COVID-19. There were recorded staff, PPE, and resource shortages in cancer facilities [68]. Disruption in the supply chain of medications and equipment contributed to delays in screening, diagnosis and treatment [69]. In many countries, cancer centres were restructured to provide care to patients with COVID-19. There were recorded staff, PPE and resource shortages in cancer facilities [68]. Disruption in the supply chain of medications and equipment contributed to delays in screening, diagnosis and treatment [69].

Globally, the pandemic caused several changes in the utilisation of health services with healthcare utilisation decreasing by about a third [70]. This includes significant decreases in the total number of individuals with cancer assessing care across several countries, irrespective of income level [71,72]. Lifesaving procedures including vaccination programmes and cancer interventions were suspended and/or delayed and the impact of these shortfalls was worse on poor people living in LMICs [73,74]. Results from an Indian cohort study revealed that during the lockdown periods, the total number of patients registered at 41 cancer centres decreased by 54% with associated decreases in hospital admissions, cancer surgeries, radiotherapy, chemotherapy and diagnostic procedures, highlighting the widespread, global impact of these concerns [75].

As the pandemic declines and post-pandemic planning begins, cancer facilities must strategically manage the backlog of cancer services and the potential for increases in later-stage presentation of cancer due to delayed prevention and diagnosis during the pandemic. This requires a multidisciplinary approach to decrease the burden on healthcare providers, patients, and other relevant stakeholders and should be adequately phased and tailored to the needs of specific facilities.

### Resource limitations

Sub-Saharan Africa is faced with distinctive endemic public health problems, due in large part to resource scarcity. Aside from the COVID-19 pandemic, many SSA countries are prone to regional epidemics of infectious diseases like viral hemorrhagic fevers, bacterial meningitis and tuberculosis [76]. Many regions lack potable water and internet connectivity and/or have poor electricity and road networks [77,78]. There is a major gap in healthcare coverage especially in rural areas and this is associated with poor quality services [79]. For example, only half of the primary care facilities in SSA have access to clean water and adequate sanitation and only a third have access to reliable electricity [79]. Healthcare is not free in most countries and only four out of 36 countries in a survey have a health insurance coverage rate of over 20% [80]. Also, less than 2% of the drugs and supplies consumed in SSA are produced within Africa, therefore, there is a significant shortage, and where available, many people are unable to afford them [81].

Many countries in SSA have a weak healthcare system. Sub-Saharan Africa contributes up to 25% of the global disease burden and has only 3% of the world’s health manpower [82]. Healthcare allocation in this region has been low and this directly impacts the quality of healthcare services provided [83]. There is less healthcare development leading to many unfunded or under-funded healthcare facilities [83]. Other challenges facing the healthcare systems within SSA include lack of appropriate technology, and centralisation of healthcare centres, lack of adequate human resources, frequent breakdown of equipment and lack of medical and diagnostic supplies among others [83]. Added to these existing challenges, the COVID-19 pandemic has contributed to the stretching of already scarce resources in this region, exacerbating pre-existing limitations on cancer care services.

### Innovations implemented

It is worth mentioning that many countries implemented innovations to fill the gaps created by the pandemic. These included altered fractionation of radiotherapy to decrease overall treatment time [45], the use of virtual platforms for consultation, follow-up, and treatment, the use of courier services and/or drones for sample collection and delivery of medications, provision of transport services and/or reimbursement and home-based self-sampling for HPV testing [10,52,55].

### Telemedicine

The use of telemedicine in patient management, through widespread deployment of virtual consultations, remote follow-up visits, virtual management team meetings and remote patient monitoring decreased the impacts of the pandemic and provided a solution to pre-pandemic location barriers for patients [10,26]. It also provided the opportunity for continued patient management by eliminating the need for an in-person consultation, decreasing the likelihood of disease exposure, and providing an assurance of social support to patients. The challenges of telemedicine in SSA include poor internet availability, especially in rural areas, power outages, costs of internet access, and patient and healthcare personnel preferences [84].

Despite these challenges, the use of telemedicine is feasible and can provide long-term benefits for cancer management, although further research is needed to explore facilitators in SSA. The potential for expansion with investment in access to the internet and technology may provide long-standing improvements for triaging patients for whom long-distance travel is required and those for whom virtual consultation and follow-up are possible. While awaiting robust internet, it may be possible to conduct teleconsultations with specialists in tertiary centres at rural primary medical homes to decrease logistical burdens and expand access. To increase acceptability among healthcare personnel, there is a need for training, and the inclusion of multilanguage support [85]. Also, healthcare leaders and policymakers must increase budgetary allocations in order to support the technology required for telemedicine [85]. The cost internet can be subsidised to increase reach especially among those living in rural areas.

### Hypofractionated and ultrahypofractionated radiotherapy

The use of hypofractionated and ultra hypofractionated radiotherapy, treatment fractionations that deliver larger doses of radiation per day. It decreases the number of treatment fractions and was essential during the pandemic as patients did not have to travel frequently during the curfews and/or lockdowns. It can reduce treatment costs, especially for patients who lost their source of income during the pandemic. It also increased patient convenience while maintaining positive outcomes [86]. If designed appropriately, it has the potential for sustainability and can alleviate burdens associated with lost wages during treatment as well as the need for long-term housing for those traveling long distances.

As services return to pre-pandemic levels, cancer facilities should prioritise adequate training of professionals, incorporate systemic changes that would result in reduced cost of care and increase funding for cancer research to improve evidence-based care. This may provide a unique opportunity to reflect on the existing cancer-directed resources and increase the provision of necessary supplies and equipment in resource-poor, overburdened centres. This would facilitate the re-escalation of not only suspended services, but also previously inadequate equipment, personnel, and volumes of supplies. Services should be offered following evidence-based guidelines to decrease infection spread. Some of the innovations discussed above have long-term benefits for improving not just cancer care but the healthcare system in SSA. However, the implementation of these innovations will require increased awareness and education, improved infrastructure including technology, and greater partnerships and collaboration within SSA.

In order to further improve cancer care in SSA, future research can focus on improving awareness of the long-term impacts of delayed cancer care. Results from such studies can be useful in increasing monetary allocations to cancer centres. To further explore the usefulness of telemedicine, research can focus on understanding the barriers and facilitators of telemedicine from the perspective of healthcare providers. The importance of other innovations can be further studied to maximise their benefits and improve the outcomes of cancer care within SSA.

### Strengths and limitations

This study is strengthened by the specificity of the research question. To the best of our knowledge, no study systematically describes the impact of the pandemic on cancer services in SSA, nor the innovations deployed to respond. This review utilised a rigorous search and data extraction process with strict eligibility criteria for selecting articles. Each step of the review methodology is reproducible using the same search and analytical methods. Further, the use of the alluvial chart to graphically show the findings enhance a wider dissemination of our results and enable different stakeholders to easily visualise our findings with a focus on finding trends across all studies.

However, many of the articles were perspectives and descriptive studies which can introduce informational and/or subjective biases and lack the rigor of epidemiological studies. This may impact some of the conclusions drawn by the researchers. Descriptive studies also provided a limited scope, with findings limited to one setting and may not be generalisable to other populations or settings within SSA. Further the trends across studies suggest that many studies did not report impact on key outcome measures (preventive services, diagnosis, and treatment), which warrant more work to fully dissect the long-term impacts of the COVID-19 pandemic on cancer care in SSA.

## CONCLUSION

The COVID-19 pandemic caused disruptions that considerably impacted cancer care in SSA. While the extent of the impact the pandemic had on cancer care is not fully understood, the implications of COVID-19 interruptions will have lasting consequences on cancer care. It is worth mentioning that steps were taken to implement innovations to mitigate the impact of the pandemic and these innovations have the potential to address challenges, including those pre-dating the pandemic, to an overburdened cancer control system in SSA. As services return to pre-pandemic status, cancer centres in SSA must focus on expanding existing infrastructure and technology to organise cancer care. This includes training for professionals, allocating resources for research on cancer care, equipping cancer centres with the necessary technology required to deliver quality care and system changes to address barriers to care. Adopting innovations can provide long-term benefits to cancer care and extend the reach of tertiary centres into the community. Government and care facilities should encourage best practices within limited resource settings and encourage multidisciplinary collaborations to improve cancer care services and research. Future research is warranted to fully dissect the long-term impact of the COVID-19 impact on cancer care, with a focus on less reported outcomes that were identified in our systematic review, including preventive services, diagnosis, and follow-up care. Delays and suspension of services might imply worse cancer outcomes in the long run.

## Additional material


Online Supplementary Document
