# Supplementary material for: The impact of the COVID-19 pandemic on cancer care including innovations implemented in Sub-Saharan Africa: A systematic review
Source: J Glob Health. 2023 Nov 17;13:06048. doi: 10.7189/jogh.13.06048 (PMC10656081; doi:10.7189/jogh.13.06048)
Supplement: Online Supplementary Document [file jogh-13-06048-s001.pdf]

Online Supplementary Document

Abunike et al. The Impact of the COVID-19 Pandemic on Cancer care including innovations implemented in Sub-Saharan Africa: A Systematic Review

Supplementary Table 1: List of MeSH terms and Keywords

MeSH terms	Keywords
“COVID-19”	SARS-CoV-2
“Africa South of the Sahara”	Coronavirus
“Neoplasms”	Subsaharan Africa
	Sub-Saharan Africa
	Cancer
	Tumor
	Tumour
	Malignancy
	Malignancies
	Carcinoma

Supplementary Table 2: Excel dataset used for Alluvial Chart

Author	Country	Cancer type	Preventive Services	Diagnosis	Treatment	Follow up
Abila et al., 2020	Uganda	Not reported	Suspended	Not reported	Delayed	Delayed
Chu et al., 2021	South Africa	Breast	Not reported	Not reported	Delayed	Not reported
Chu et al., 2021	South Africa	Colorectal	Not reported	Not reported	No Change	Not reported
Davey et al., 2021	Botswana	Gynecologic	Not reported	Delayed	Not reported	NCNP
Davey et al., 2021	Botswana	Gynecologic	Not reported	Delayed	Not reported	SOP
Benn et al., 2021	South Africa	Breast	Not reported	Not reported	Delayed	Not reported
Desta et al., 2021	Ethiopia	Cervical	Delayed	Delayed	Delayed	Not reported
Ezenwankwo et al., 2021	Multiple	Prostate	Not reported	Delayed	Delayed	Not reported
Ezenwankwo et al., 2022	West Africa	Prostate	Suspended	Delayed	Delayed	Not reported
Haddison et al., 2022	Cameroon	Cervical	Delayed	Not reported	Not reported	Not reported
Henke., 2021	Multiple	Not reported	Suspended	Delayed	Delayed	Not reported
Joseph et al., 2022	Nigeria	Not reported	Not reported	Not reported	Delayed	Not reported
Karanja-Chege., 2022	Kenya	Cervical	Suspended	Not reported	Not reported	Not reported
Lombe et al., 2020	Zambia	Not reported	Not reported	Not reported	Delayed	Delayed
Martei et al., 2021	Multiple	Not reported	Not reported	Not reported	Delayed	Not reported
Masege et al., 2020	South Africa	ENT	Not reported	Delayed	Not reported	Not reported
Moustakis et al., 2020	South Africa	Not reported	Not reported	Not reported	Delayed	Not reported
Murewanhema., 2021a	Zimbabwe	Cervical	Suspended	Not reported	Not reported	Not reported
Murewanhema., 2021b	Zimbabwe	Cervical	Not reported	Not reported	Delayed	Not reported
Murewanhema et al., 2020	Zimbabwe	Not reported	Not reported	Not reported	Suspended	Suspended
Olabumuyi et al., 2020	Nigeria	Not reported	Not reported	Not reported	Suspended	Not reported
Ramdas et al., 2020	South Africa	Breast	Not reported	Not reported	No Change	Not reported
Villain et al., 2021	Rwanda	Not reported	No Change	No Change	No Change	Not reported
Villain et al., 2021	Cote d'Ivoire	Not reported	No Change	No Change	No Change	Not reported
Villain et al., 2021	Cameroon	Not reported	Suspended	No Change	Suspended	Not reported
Villain et al., 2021	Zambia	Not reported	Suspended	No Change	No Change	Not reported
Van Wyk et al., 2021	South Africa	Breast	Not reported	Delayed	Not reported	Not reported
Van Wyk et al., 2021	South Africa	Cervical	Not reported	Delayed	Not reported	Not reported

Umutesi et al., 2021	Rwanda	Not reported	Suspended	Suspended	Suspended	Not reported
Umar et al., 2022	Kenya	Not reported	Not reported	Delayed	Delayed	Not reported
Sormani et al., 2021	Cameroon	Cervical	Suspended	Not reported	Not reported	Not reported
Okunade et al., 2020	Nigeria	Gynecologic	Not reported	Delayed	Delayed	Not reported
Kugbey et al., 2020	Ghana	Not reported	Not reported	Delayed	Delayed	Not reported
Salako et al., 2020	Nigeria	Not reported	Not reported	Not reported	Suspended	Not reported
Vanderpuye et al., 2020	West Africa	Not reported	Not reported	Not reported	Suspended	Not reported
Vanderpuye et al., 2020	South Africa	Not reported	Not reported	Not reported	No Change	Suspended

Quality Assessment

Supplementary Table 3: Newcastle - Ottawa Quality Assessment Scale for Cross-Sectional Studies

**Author**		**Representativeness of the cases**	**Sample size**	**Non-Response rate**	**Ascertainment of the screening/surveillance tool**	**Comparability**	**Assessment of the outcome**	**Statistical test**	**Total score (Maximum 10)**	**Overall Quality Assessment**
Chu et al, 2020	**SAA**	*	*	-	-	-	*	*	4*	Average = 4* Poor
	**RCR**	*	*	-	-	-	*	*	4*	
Haddison et al, 2022	**SAA**	*	*	*	*	-	*	*	6*	Average = 5.5* Fair
	**RCR**	*	-	*	*	-	*	*	6*	
Irusen et al, 2021	**SAA**	*	*	*	**	-	*	*	**7***	Average = 6.5* Good
	**RCR**	-	*	*	**	-	*	*	6*	
Joseph et al, 2022	**SAA**	*	*	-	*	-	*	*	5*	Average = 5.5* Fair
	**RCR**	*	*	*	*	-	*	*	6*	
Martei et al, 2021	**SAA**	*	-	-	*	-	*	*	4*	Average = 3.5* Poor
	**RCR**	-	-	-	*	-	*	*	3*	
Villain et al, 2021	**SAA**	*	*	*	*	-	*	*	6*	Average = 5* Fair
	**RCR**	-	-	*	*	-	*	*	4*	
Umar et al, 2022	**SAA**	*	*	*	*	-	*	*	6*	Average = 6* Good
	**RCR**	*	*	*	*	-	*	*	6*	
Sikakulya et al, 2021	**SAA**	*	*	-	*	-	*	*	5*	Average = 5* Fair
	**RCR**	*	*	-	*	-	*	*	5*	

Studies with a total score of 8 points and above were considered to have a low risk of bias; 5 to 7 points were considered to have a medium risk of bias; less than 5 points were considered to have a high risk of bias.

Supplementary Table 4: Newcastle - Ottawa Quality Assessment Scale for Cohort Studies

**Author**	**Commenters**	**Representativeness of the exposed cohort**	**Selection of non-exposed**	**Ascertainment of exposure**	**Outcome not at study start**	**Comparability**	**Assessment of outcome**	**Length of follow-up**	**Adequacy of follow-up**	**Total score**	**Overall quality assessment**
**Argefa & Roets, 2021**	**SAA**	*		**	*		*	*	*	**7***	Good
	**RCR**	*		**	*		*	*	*	**7***	Good
										**Average score = 7***	Good

Studies with a total score of 8 points and above were considered to have a low risk of bias; 5 to 7 points were considered to have a medium risk of bias; less than 5 points were considered to have a high risk of bias.

Supplementary Table 5: Quality Assessment Tool for Before – After (Pre-Post) Studies with No Control Group

The quality assessment tool for before-after (pre-post) studies with no control group: scores of included studies. Adopted from the National Heart, Lung, and Blood Institute. Available on: https://www.nhlbi.nih.gov/health-topics/study-quality-assessment-tools

**Authors**	**Reviewer**	**Scale Items**												**Score**
Desta et al, 2021		**1**	**2**	**3**	**4**	**5**	**6**	**7**	**8**	**9**	**10**	**11**	**12**	

	**SAA**	Y	Y	Y	CD	NA	Y	Y	CD	CD	Y	N	CD	Fair
	**RCR**	Y	Y	Y	CD	NA	Y	Y	CD	CD	Y	CD	CD	Fair
Diao et al, 2022		**1**	**2**	**3**	**4**	**5**	**6**	**7**	**8**	**9**	**10**	**11**	**12**	
	**SAA**	Y	CD	Y	Y	N	Y	Y	CD	Y	N	CD	NA	Fair
	**RCR**	Y	Y	Y	Y	N	Y	Y	CD	Y	CD	N	NA	Good
Muli et al, 2021		**1**	**2**	**3**	**4**	**5**	**6**	**7**	**8**	**9**	**10**	**11**	**12**	
	**SAA**	Y	Y	Y	Y	Y	Y	Y	N	CD	N	N	N	Good
	**RCR**	Y	Y	Y	Y	Y	Y	Y	N	CD	N	N	N	Good

Refer to the table below for the criteria

**Criteria**	**Scale Items**
Was the study question or objective clearly stated?	1
Were eligibility/selection criteria for the study population pre-specified and clearly described?	2
Were the participants in the study representative of those who would be eligible for the test/service/intervention in the general or clinical population of interest?	3
Were all eligible participants that met the pre-specified entry criteria enrolled?	4
Was the sample size sufficiently large to provide confidence in the findings?	5
Was the test/service/intervention clearly described and delivered consistently across the study population?	6
Were the outcome measures pre-specified, clearly defined, valid, reliable, and assessed consistently across all study participants?	7
Were the people assessing the outcomes blinded to the participants' exposures/interventions?	8
Was the loss to follow-up after baseline 20% or less? Were those lost to follow-up accounted for in the analysis?	9

Did the statistical methods examine changes in outcome measures from before to after the intervention? Were statistical tests done that provided p values for the pre-to-post changes?	10
Were outcome measures of interest taken multiple times before the intervention and multiple times after the intervention (i.e., did they use an interrupted time-series design)?	11
If the intervention was conducted at a group level (e.g., a whole hospital, a community, etc.) did the statistical analysis take into account the use of individual-level data to determine effects at the group level?<br>*If this question is not applicable, the total score is out of 11, not 12.	12

Key: Y = Yes, N = No, NR = Not reported, CD = Cannot determine, NA = Not applicable

Supplementary Table 6: Quality Assessment for Perspective, Descriptive, and Retrospective Studies

**Article**	**SAA comments**	**RCR comments**	**Conflict of interest (Noted)**
Abila et al, 2020	This expert opinion is subjective and prone to informational bias. May have limited scope and poor generalizability.	Expert opinion only reflects the opinion(s) of the author; therefore, generalizations may not be conclusive.	No COI cited
Bhutta et al, 2021	This descriptive study has a limited scope and cannot be generalized to other settings	A descriptive study that conducted in a single area, generalizations may be impacted.	No COI cited
Chu et al, 2021	In this retrospective study, poorly stored data can affect the quality of results.	A retrospective study whose results and conclusions may be impacted by poor data storage. Study	No COI cited

	Findings may not be generalizable.	conducted in one site limiting generalizability.	
Davey et al, 2021	This descriptive study is prone to lack of generalizability, with limited scope.	A descriptive study that could be prone to subjective bias and generalizability is limited.	6th author: Roche/Genentech, Novartis, MSD, Bristol-Myers Squibb, Pfizer, Mundifarma, AstraZeneca.<br>8th author: Pfizer, MSD, Mafemi PTY LTD, VIA Global Health
Benn et al, 2021	A letter to the editor is subjective and prone to informational bias.	A letter to the editor reflects the opinion(s) of the author, therefore prone to subjective and informational bias with limited generalizability.	BLR reports personal fees, advisory boards, and speaker engagements from Novartis South Africa. Personal fees, advisory boards, and speaker engagements from Elli-Lilly South Africa. Personal fees, advisory boards, and speaker engagements from AstraZeneca South Africa during the conduct of the study.
Ezenwankwo & Nnaji, 2021	A descriptive study that is prone to informational bias, is subjective and non-generalizable. Reflecting mostly the author's opinions within a specific context only.	Prone to subjectivity and reflects only the opinion(s) of the authors.	No COI cited
Ezenwankwo et al, 2022	This perspective piece is prone to subjectivity and the findings cannot be generalized to other contexts.	Prone to subjectivity and reflects only the opinion(s) of the authors.	No COI cited
Henke, 2021	This letter to the editor reflects the author's opinions within a particular context, therefore, the reports may not be generalizable and prone to subjective bias.	Limited to the opinions of the author. Lacks rigor and cannot be generalized.	No COI cited

Karanja-Chege, 2022	This review provides a description within a specific context. This impacts generalization and is subjected to biases.	Captures events within a specific context. It has limited generalizability.	No COI cited
Lombe et al, 2020	The article provides a description of services in one country. Findings are non-generalizable, and the author’s opinions could be subjective.	Findings are limited to one setting, this impacts generalization of findings.	No COI cited
Masege et al, 2020	Description is limited to one facility, in one country. This may not be generalizable. Poor record keeping could impact results.	Findings are limited to one facility in a single country. Results may not be extrapolated to other settings.	No COI cited
Moustakis et al, 2020	This retrospective study may be affected by inadequate record-keeping. Records were obtained from one facility which impacts generalizability.	Prone to selection bias while choosing sample. Poor record keeping can impact quality of results and findings.	No COI cited
Murewanhema, 2021	This perspective study may be subjective and prone to informational bias, with limited generalizability	Limited to the opinions of the author therefore, findings may not be reflective of the experiences in other settings.	No COI cited
Murewanhema, 2021	This perspective and recommendation article has limited generalizability. The contexts of the report are limited to one setting.	Limited to the opinions of the author therefore, findings may not be reflective of the experiences in other settings.	
Murewanhema, 2020	This perspective and recommendation article has limited generalizability. The contexts of the report are limited to one setting.	Limited to the opinions of the author therefore, findings may not be reflective of the experiences in other settings.	No COI cited
Ngwa et al, 2022	The findings reported may be generalizable to many settings within SSA. Informational	Involved experts from different SSA countries. Therefore, there is a decrease in	JD and KG received grants or contracts from Takeda Pharmaceutical, Johnson and Johnson,

	bias may be minimal as the commission responsible for the study was made up of experts from different SSA countries.	informational bias. Also, the findings are reflective of experiences of more than one country.	AstraZeneca, Cepheid, Merck, and Pfizer as funding for specific cancer projects. JG has served as the previous chair of the South African National Advisory Committee on Cancer Control<br><br>OWB received a P30 National Cancer Institute cancer grant to Johns Hopkins University. And has had a leadership or fiduciary role paid or unpaid at Lyell Immunopharma, PDS Biotech, and Grail. Also has stock or stock options with Lyell Immunopharma and PDS Biotech.<br><br>AR received the Schneider-Lesser award as a junior faculty grant<br><br>TRR served as principal investigator on grants from the National Cancer Institute including P20 CA233255, R01CA207365, and U01CA184374. And received consulting fees from serving on 13 US cancer center scientific advisory boards.
Olabumuyi et al, 2020	Expert opinions may be biased by the limitations of the expert. Also, experts may be biased to elaborate their belief systems which may	Expert opinions are not rigorous. It reflects only the opinions of the experts which may differ from one individual to another.	No COI cited

	differ from existing trends.		
Osibogun et al, 2021	The results of this retrospective study could be impacted by poor record keeping. Records were obtained from one setting which impacts generalizability.	Prone to selection bias. Also, incomplete data collection and poor record keeping can impact quality of result.	No COI cited
Ramdas et al, 2020	With retrospective studies, certain important details could be missed from the medical record. Data is collected from a single setting which can impact generalizability.	Incomplete data impacts quality of findings. Also, samples are drawn from a single setting, this limits generalizability of findings.	YR: Consulting or advisory role with Norvatis<br><br>MVH: Employment with Netcare.<br><br>No other COI cited
Salcedo et al, 2021	Descriptive review is limited to a single setting. Generalizability can be impacted.	Description of experiences in a single setting. Findings cannot be generalized.	No COI cited
Weber et al, 2021	The authors described the outcome of an intervention. It is prone to a lack of generalizability since the intervention occurred in one center.	Intervention carried out in a single setting. The outcomes may not be achieved in other settings.	No COI cited
Van Wyk et al, 2021	This retrospective analysis is prone to errors if medical records were collected and stored properly. Also lacks generalizability.	Incomplete data impacts quality of findings. Also, samples are drawn from a single setting, this limits generalizability of findings.	No COI cited
Umutesi et al, 2021	This descriptive review is limited to one setting, which impacts generalizability. Also, subject to the author's opinions and experiences.	Reflects opinions of the authors therefor prone to subjective bias. Lacks rigor and findings may not be generalizable.	No COI cited
Sumbana et al, 2020	This descriptive review is limited to one setting, which impacts generalizability. Also, subject to the author's opinions and experiences.	Provides summary of experiences in a single setting. Prone to subjective and informational bias.	No COI cited

Sormani et al, 2021	This descriptive review provides only a summary of the authors' experiences. Causal relationships cannot be established, and generalizability is limited.	Provides summary of experiences in a single setting. Prone to subjective and informational bias.	
Okunade et al, 2020	This perspective and recommendation reflect the opinions of the authors which may be subjective and prone to informational bias. The recommendations may not be generalizable to other settings.	Reflects the opinions of the authors. The recommendations may only apply to a single setting with limited generalizability.	No COI cited
Selgado et al, 2021	Retrospective analysis is prone to errors if data collection is inadequate. Causal relationships are not readily established, and findings have limited generalizability.	Data collected from one setting: this impacts generalizability. Also, incomplete data impacts quality of findings.	No COI cited
Rosa et al, 2021	Descriptive study limited to a single setting, prone to informational bias and limited generalizability.	Prone to informational and subjective biases. Limited generalizability.	No COI cited
Kugbey et al, 2020	This descriptive study has limited generalizability as findings were obtained from one setting. Also, the findings may be subjective to authors' opinions and prone to informational bias.	Prone to informational and subjective biases. Limited generalizability.	No COI cited
Kassaman et al, 2020	This descriptive review is limited to one setting, which impacts generalizability. Also, subject to the author's opinions and experiences.	Prone to informational and subjective biases. Limited generalizability.	No COI cited
Salako et al, 2020	The article provides a description of services in one country. Findings are non-	Findings from a single setting which impacts generalizability. Prone to informational bias.	No COI cited

	generalizable, and the author’s opinions could be subjective.		
Okeke et al, 2020	This perspective article may have wider generalizability. The context of the report explores more than one setting but may be based on the opinions and experiences of the authors.	Reflects the opinions and experiences of the authors, increasing subjective and informational biases.	No COI cited
Vanderpuye et al, 2020	The perspective piece reflects the experiences and opinions of the authors. Therefore, findings may not be generalizable.	The perspective piece reflects the experiences and opinions of the authors. Therefore, findings may not be generalizable.	No COI cited
